# The Impact of Gastroesophageal Disease on Clostridium difficile Infection Hospitalization: A Nationwide Analysis From the United States

**DOI:** 10.7759/cureus.62223

**Published:** 2024-06-12

**Authors:** Sajana Poudel, Manoj Ghimire, Ayusha Poudel, Kalpana Ghimire, Karun Shrestha, Prakriti Subedi, Sumina Rai

**Affiliations:** 1 Internal Medicine, John H. Stroger, Jr. Hospital of Cook County, Chicago, USA; 2 Internal Medicine, St. Barnabas Hospital, New York, USA

**Keywords:** diarrhea, acid suppression, gastroesophageal reflux disease (gerd), national inpatient sample, clostridium difficile

## Abstract

Background

Enterocolitis due to *Clostridium difficile* infection (CDI) is one of the most common infectious causes of healthcare-associated diarrhea and a significant cause of morbidity and mortality among hospitalized patients. Gastroesophageal reflux disease (GERD) is notable for its high prevalence, variety of clinical presentations, and underrecognized morbidity. It is widely treated with acid suppression, both with over-the-counter and prescription medications. There are no studies evaluating the impact of GERD on CDI hospitalization. In this study, we aimed to analyze the influence of concomitant GERD on patients hospitalized for CDI enterocolitis.

Methodology

This was a retrospective, observational study where we extracted data from 2016 to 2020 from the National Inpatient Sample database. We included all patients hospitalized with a primary discharge diagnosis of CDI with or without a secondary diagnosis of GERD. We compared the demographics, comorbidities, and in-hospital outcomes between these two groups.

Results

This study identified 239,603 hospitalizations with a discharge diagnosis of CDI. Of these, 67,000 (28%) had a concurrent diagnosis of GERD. Patients with GERD had a higher prevalence of hypertension (41% vs. 35.5%, p < 0.01), hyperlipidemia (50% vs. 36.5%, p < 0.01), obesity (13.7% vs. 10.5%, p < 0.01), coronary artery disease (24.4% vs. 19.6%, p < 0.01), and chronic kidney disease (20.7% vs. 19.2%, p < 0.01). Notably, inpatient mortality was lower in CDI hospitalizations with GERD (0.66% vs. 1.46%, p < 0.01). The total hospital charge was reduced in the CDI with GERD group in comparison to the CDI without GERD group (39,599 vs. 43,589, p < 0.01). The length of hospital stay was similar between the two groups (5.3 vs. 5.4 days, p = 0.07). Regarding complications, CDI hospitalizations with GERD demonstrated lower rates of hypovolemic shock (0.5% vs. 0.73%, p = 0.06), septic shock (0.6% vs. 1.05%, p < 0.01), acute kidney injury (1.48% vs. 2.04%, p < 0.01), intestinal perforation (0.008% vs. 0.16%, p = 0.03), and lactic acidosis (0.008% vs. 0.16%, p = 0.03). Conversely, CDI patients with GERD had a higher rate of ileus (2.66% vs. 2.16%, p < 0.01).

Conclusions

Patients with CDI and concurrent GERD exhibited favorable in-hospital outcomes in terms of complication rates, mortality, and total hospital charges. Further research is required to comprehensively explore and validate these findings.

## Introduction

Enterocolitis due to *Clostridium difficile* infection (CDI) is one of the most prevalent causes of healthcare-associated infectious diarrhea, significantly contributing to morbidity and mortality among hospitalized patients [1]. Traditionally, CDI predominantly affects older patients with healthcare exposure; however, its incidence is rising among younger individuals and within community settings [2-5].

Some of the risk factors for CDI are antibiotic exposure, hospitalization, serious underlying illnesses, immunocompromising conditions, and advanced age. There is also an interesting association between gastric acid suppression and CDI [6].

Gastroesophageal reflux disease (GERD) is characterized by its high prevalence, diverse clinical presentations, underrecognized morbidity, and substantial economic impact. A systematic review estimated the prevalence of GERD in the United States to be between 18.1% and 27.8% [7]. Given the substantial population treated with acid suppression for GERD, these individuals are at an elevated risk for CDI, as acid suppression is a well-established risk factor for CDI [8]. Consequently, understanding the impact of GERD on in-hospital outcomes related to CDI is crucial.

To date, no studies have specifically investigated the outcomes of CDI in patients with GERD. This study aimed to explore how the presence of GERD influences in-hospital outcomes among individuals with CDI. Additionally, we examined the demographic characteristics of CDI hospitalizations with and without concomitant GERD to identify potential risk factors and patterns associated with these populations.

## Materials and methods

Study population and design

A retrospective, observational study was conducted using the National Inpatient Sample (NIS) database. Data were obtained regarding demographics, primary and secondary diagnosis, in-hospital complications, mortality, hospital length of stay (LOS), and total hospital charges. The data were obtained from the Healthcare Cost and Utilization Project database from 2016 to 2020. Ethics committee approval was not necessary as the NIS database is exempt from institutional review board approval. The datasets are publicly available in the data repository at https://www.hcup-us.ahrq.gov/databases.jsp.

Our study identified 239,603 hospital discharges between 2016 and 2020, who were older than 18, with a discharge diagnosis of CDI with or without GERD. We used the following International Classification of Diseases, 10th Revision, Clinical Modification (ICD-10-CM) codes: enterocolitis due to *Clostridium difficile* (A04.7) and GERD (K21.00, K21.01, K21.9) to obtain the data.

Primary and secondary outcomes

The primary outcomes of this study were mortality, hospital LOS, and total hospital charges. Secondary outcomes of the study were various hospital complications such as sepsis, renal failure, and intestinal perforation.

Statistical analyses

Patient information and data were analyzed using Stata version 17 (StataCorp., College Station, TX, USA). Chi-square and Student’s t-tests were used to compare categorical and continuous variables, respectively. Frequencies or confidence intervals, and p-values were reported for all outcomes with the statistical significance level set at a p-value <0.05.

## Results

From 2016 to 2020, we identified 239,603 hospital discharges of CDI, including both first and recurrent episodes. Of these hospitalizations, 67,000 (28%) had a concurrent secondary diagnosis of GERD. For CDI hospitalizations for both with and without GERD, the majority were Caucasians, followed by African Americans, Hispanics, and other races. In CDI hospitalizations with GERD, we observed a higher percentage of patients with comorbidities compared to those without GERD, including hypertension (41% vs. 35.5%, p < 0.01), hyperlipidemia (50% vs. 36.5%, p < 0.01), obesity (13.7% vs. 10.5%, p < 0.01), coronary artery disease (24.4% vs. 19.6%, p < 0.01), and chronic kidney disease stages I to IV (20.7% vs. 19.2%, p < 0.01). A higher proportion of CDI hospitalizations for both with and without GERD were noted in the South region, followed by the Midwest, Northeast, and West (Table 1).

**Table 1:** Demographic characteristics of the study population. *: significant p-value. GERD = gastroesophageal reflux disease; CI = confidence interval

Patient characteristics	With GERD (n = 67,000)	Without GERD (n = 172,603)	P-value
Age (years), mean (95% CI)	67.8 (67.6-68.1)	66.1 (65.9-66.3)	<0.01*
Gender, n (%)			
Female	45,828 (68.4)	107,704 (62.4)	<0.01*
Race/Ethnicity, n (%)			
White	54,203 (80.9)	131,005 (75.9)	<0.01*
Black	6,767 (10.1)	19,849 (11.5)	
Hispanic	4,013 (5.99)	14,326 (8.3)	
Asian	469 (0.7)	2,243 (1.3)	
Native American	335 (0 5)	1,035 (0.6)	
Others	1,005 (1.5)	3,624 (2.1)	
Hospital size, n (%)			
Small	16,147 (24.1)	41,252 (23.9)	0.75
Medium	19,698 (29.4)	50,227 (29.1)	
Large	31,088 (46.4)	80,778 (46.8)	
Hospital region, n (%)			
Northeast	12,864 (19.2)	32,449 (18.8)	<0.01*
Midwest	18,425 (27.5)	40,906 (23.7)	
South	27,001 (40.3)	67,832 (39.3)	
West	8,576 (12.8)	31,068 (18)	

CDI hospitalizations with GERD had lower inpatient mortality than those without GERD (0.66% vs. 1.46%, p < 0.01). Total hospital charges were lower in the CDI with GERD (39,599 vs. 43,589, p < 0.01). Hospital LOS between the two groups was similar (5.3 vs. 5.4 days, p = 0.07). Regarding complications, CDI patients with GERD had lower rates of hypovolemic shock (0.5% vs. 0.73%, p < 0.01), septic shock (0.6% vs. 1.05%, p < 0.01), acute kidney injury (1.48% vs. 2.04%, p < 0.01), intestinal perforation (0.008% vs. 0.16%, p = 0.03), and lactic acidosis (0.008% vs. 0.16%, p = 0.03). However, patients with GERD had a slightly higher rate of ileus (2.66% vs. 2.16%, p < 0.01) (Table 2).

**Table 2:** Clinical characteristics and outcomes of the study population. *: significant p-value. GERD = gastroesophageal reflux disease; LOS = length of stay; CI = confidence interval

Clinical characteristics	With GERD (n = 67,000)	Without GERD (n = 172,603)	P-value
Comorbidities, n (%)			
Diabetes	6,566 (9.8)	16,397 (9.5)	0.39
Hypertension	27,872 (41.6)	61,274 (35.5)	<0.01*
Heart failure	12,931 (19.3)	31,120 (18.03)	<0.01*
Chronic kidney disease	13,869 (20.7)	33,139 (19.2)	<0.01*
Hyperlipidemia	33,500 (50)	63,000 (36.5)	<0.01*
Coronary artery disease	16,348 (24.4)	33,830 (19.6)	<0.01*
Obesity	9,179 (13.7)	18,123 (10.5)	<0.01*
Chronic obstructive pulmonary disease	335 (0.5)	1,260 (0.73)	<0.01*
Alcohol abuse	1,121 (1.66)	3,262 (1.89)	0.09
Smoking	650 (0.97)	1,829 (1.06)	0.35
Complications, n (%)			
Hypovolemic shock	335 (0.5)	1,260 (0.73)	<0.01*
Acute kidney injury	985 (1.48)	3,540 (2.04)	<0.01*
Intestinal perforation	55 (0.008)	280 (0.16)	0.03*
Lactic acidosis	55 (0.008)	280 (0.16)	0.03*
Septic shock	400 (0.6)	1,820 (1.05)	<0.01*
Outcomes			
Mortality, n (%)	100 (0.66)	590 (1.46)	<0.01*
LOS in days, mean (95% CI)	5.3 (5.2-5.4)	5.4 (5.3-5.5)	0.02*
Total hospital charges, $, mean (95% CI)	39,599 (38,633-40,564)	43,589 (42,737-44,440)	<0.01*

## Discussion

The findings from our study highlight the potential impact of concomitant GERD on in-hospital outcomes among individuals hospitalized with CDI.

The average age for patients admitted with CDI as a principal diagnosis in our study was 66.5 years, consistent with results reported by other studies [4]. Our study found a higher prevalence of CDI among Caucasian patients in both groups. This result may be attributed to greater access to healthcare among the Caucasian population. A NIS study by Mao et al. showed similar racial differences in CDI hospitalizations [9]. The comparison between CDI rates in hospitalized Caucasian individuals and those of other races indicates that any disparities in CDI risk are probably due to unequal access to healthcare, rather than differences in genetics [9].

CDI hospitalizations in individuals with GERD demonstrated a significantly lower inpatient mortality rate compared to those without GERD (0.66% vs. 1.46%, p < 0.001). No studies have been conducted to observe the outcome of CDI hospitalizations in patients with GERD. GERD is usually treated with acid suppression which can be available easily over the counter or via prescription. A systematic analysis found an increased risk of CDI (odds ratio (OR) = 1.74, 95% confidence interval (CI) = 1.47-2.85) or risk of recurrent CDI (OR = 2.51, 95% CI = 1.16-5.44) with long-term proton pump inhibitor (PPI) use [10].

Our study suggested a potential protective effect of GERD against CDI-related mortality, warranting further investigation into the mechanisms underlying this association. PPIs, for example, are known to alter gastric pH, which may indirectly impact the gut microbiota and potentially reduce the risk of CDI severity and mortality. However, it is important to note that while PPIs are effective in reducing gastric acid secretion, they have also been associated with increased susceptibility to enteric infections, including CDI. Therefore, the exact role of acid-suppressive medications in the context of CDI mortality warrants further investigation.

The observed lower total hospital charges among CDI patients with GERD may be attributed to differences in disease severity, resource utilization, and healthcare management strategies between the two groups. Additionally, the potential protective effect of GERD against CDI-related complications, as evidenced by the lower rates of hypovolemic shock, septic shock, acute kidney injury, intestinal perforation, and lactic acidosis, may have contributed to the overall reduction in healthcare expenditures among this subgroup.

CDI hospitalizations with GERD had higher comorbidities such as obesity, hypertension, hyperlipidemia, coronary artery disease, and chronic kidney disease. It is well known that GERD is more prevalent in obese individuals, and obesity is one of the major risk factors for high blood pressure, hyperlipidemia, and other heart diseases. Obesity is also known to be associated with an increased risk of GERD; studies have found the incidence of GERD in the United States to be between 18.1% and 27.8%, with an OR of 1.58 for having GERD in obese individuals [11].

One of the limitations of our study was that we did not categorize the severity of CDI or GERD which might be confounding in the outcomes we observed. Another limitation is the absence of an ICD-10 code for acid suppression therapy, a factor that could influence CDI risk and act as a potential confounding variable. Other limitations include the inability to include specific laboratory values, different medications used, and other specific clinical interventions as these variables cannot be obtained in detail from NIS data. Further, the accuracy of NIS data depends on accurate coding by healthcare providers and health institutions which might make it prone to error. Despite these limitations, our study is based on a large nationwide population, and it can be applied to different healthcare settings. It contributes to the existing body of literature on CDI hospitalizations and the impact of different comorbid conditions on in-hospital outcomes.

Based on the findings and limitations of our study, future research should focus on several key areas to further explore the relationship between GERD, acid suppression therapy, and CDI outcomes. Detailed investigations into the severity of GERD and information on concurrent medications used for its control are necessary to better understand their impact on CDI. Additionally, the mechanisms underlying the potential protective effect of GERD against CDI-related mortality warrant deeper exploration. Investigating how gastroesophageal reflux alters the gut microbiota and influences CDI severity could provide valuable insights.

## Conclusions

CDI patients with GERD exhibit a lower inpatient mortality rate and an overall lower rate of in-hospital complications. The higher prevalence of comorbidities such as obesity, hypertension, and hyperlipidemia among CDI patients with GERD underscores the complex interplay between these conditions. Our findings emphasize the importance of considering GERD as a modifier of CDI outcomes and healthcare utilization.
